# Osthole confers neuroprotection against cortical stab wound injury and attenuates secondary brain injury

**DOI:** 10.1186/s12974-015-0373-x

**Published:** 2015-09-04

**Authors:** Yang Xia, Liang Kong, Yingjia Yao, Yanan Jiao, Jie Song, Zhenyu Tao, Zhong You, Jingxian Yang

**Affiliations:** Department of Engineering, University of Oxford, Oxford, OX1 3LZ UK; School of Pharmacy, Liaoning University of Traditional Chinese Medicine, Dalian, 116600 China

**Keywords:** Osthole, Cortical stab wound injury, Secondary brain injury, Anti-inflammation, Anti-apoptosis

## Abstract

**Background:**

Neuroendoscopy is an innovative technique for neurosurgery that can nonetheless result in traumatic brain injury. The accompanying neuroinflammation may lead to secondary tissue damage, which is the major cause of delayed neuronal death after surgery. The present study investigated the capacity of osthole to prevent secondary brain injury and the underlying mechanism of action in a mouse model of stab wound injury.

**Methods:**

A mouse model of cortical stab wound injury was established by inserting a needle into the cerebral cortex for 20 min to mimic neuroendoscopy. Mice received an intraperitoneal injection of osthole 30 min after surgery and continued for 14 days. Neurological severity was evaluated 12 h and up to 21 days after the trauma. Brains were collected 3–21 days post-injury for histological analysis, immunocytochemistry, quantitative real-time PCR, and terminal deoxynucleotidyl transferase dUTP nick end labeling (TUNEL) and enzyme-linked immunosorbent assays.

**Results:**

Neurological function improved in mice treated with osthole and was accompanied by reduced brain water content and accelerated wound closure relative to untreated mice. Osthole treatment reduced the number of macrophages/microglia and peripheral infiltrating of neutrophils and lowered the level of the proinflammatory cytokines interleukin-6 and tumor necrosis factor α in the lesioned cortex. Osthole-treated mice had fewer TUNEL+ apoptotic neurons surrounding the lesion than controls, indicating increased neuronal survival.

**Conclusions:**

Osthole reduced secondary brain damage by suppressing inflammation and apoptosis in a mouse model of stab wound injury. These results suggest a new strategy for promoting neuronal survival and function after neurosurgery to improve long-term patient outcome.

## Background

Neuroendoscopy is a widely used neurosurgical option that employs a cylindrical retractor to access deep intracranial lesions [1]. Both retractors and surgical procedures have been improved to facilitate and reduce the invasiveness of the procedure [2–4]; in recent years, commercial products such as BrainPath (NICO Corporation, 2014) [5] have been adopted to treat hard-to-reach areas. Nonetheless, while it is superior to traditional methods such as craniotomy, neuroendoscopy can nonetheless inflict damage to brain tissue.

During a typical neuroendoscopy procedure, mechanical trauma is caused by retractor insertion and includes the severance of capillaries, extracellular matrix, and glial and neuronal processes, leading to increased pressure region surrounding the retractor [6]. Moreover, given that the retractor rests inside the brain for the duration of the surgery, brain pulsation can cause healthy tissue to beat against the rigid surface for up to several hours, inducing further injury [7]. Finally, secondary brain injury may develop hours or even days later and is the main cause of delayed neuronal death after surgery [8, 9] and includes brain edema, reduction of regional blood flow, inflammation, apoptotic cell death, oxidative stress, and gliosis [10]. Since primary brain injury caused by retractor insertion is irreversible, the main objective of medical treatment concerns the prevention and treatment of secondary brain injury [11].

The inflammatory response is a key factor in secondary injury following brain trauma and is induced by the release of proinflammatory cytokines [12, 13] that lead to the recruitment of peripheral leukocytes to the cerebral parenchyma and activation of resident immune cells [14, 15]. Neutrophils, monocytes, and lymphocytes modulate neuronal survival and death at the site of injury [14–17], while activated microglia release cytokines, reactive oxygen species, and other cytotoxic factors, further inducing neuronal death [16–18]. This response may be mitigated by suppressing inflammation with anti-inflammatory drugs. For example, the non-steroidal anti-inflammatory drug ibuprofen inhibits inflammation in mice following brain trauma by suppressing prostaglandin synthesis via cyclooxygenase 2 [8]. Some herbs used in traditional medicine exert similar effects. Triptolide, an active ingredient of *Tripterygium wilfordii* Hook F, promotes the repair of injured spinal cord by inhibiting astrogliosis and inflammation [19], whereas the root of *Panax ginseng* C.A. Meyer (Araliaceae), also known as ginseng, inhibits interleukin (IL)-1β and IL-6, tumor necrosis factor (TNF)-α, and caspase-3 and B cell lymphoma (Bcl)-2-associated X protein (Bax) expression and stimulates IL-10, thereby suppressing inflammation and apoptotic cell death after traumatic brain injury [11].

**Fig. 1:**
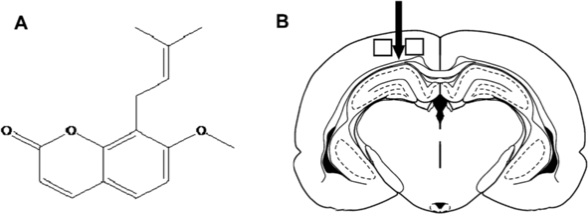
Structure of osthole and schematic illustration of a coronal brain section. **a** Chemical structure of osthole. **b** Schematic illustration of a coronal section of the mouse brain showing the relationship between the lesion cavity (*arrow*) and imaged areas (in *boxes*). Regions (500 μm^2^) immediately surrounding the cavity were selected for histopathological and immunohistochemical analyses

The natural coumarin derivative 7-methoxy-8-isopentenoxycoumarin, also known as osthole (Fig. 1), was isolated from medicinal plants such as *Cnidium monnieri* (L.) Cusson and has anti-inflammatory, anti-apoptotic, anti-oxidative stress, and neurotrophic properties that make it promising for therapeutic applications [20–23]. Osthole exerts neuroprotective effects in experimental models of cerebral ischemia/reperfusion injury via anti-oxidative and -inflammatory activities [24], inhibits immune diseases such as arthritis and hepatitis via modulation of inflammatory cytokines [25–27], and attenuates central nervous system inflammation and demyelination in experimental autoimmune encephalomyelitis (EAE) by preventing the reduction in nerve growth factor while suppressing interferon (IFN)-γ level [28]. Our previous studies have shown that osthole (30 mg/kg by intraperitoneal (i.p.) injection for 50 days) protects neurons and oligodendrocytes from inflammation-induced damage and promotes their survival and also improves the survival of engrafted neural stem cells and induces remyelination and axonal growth in EAE mice [20].

Based on the above findings, we hypothesized that osthole can confer neuroprotection and attenuate the inflammatory response and reduce secondary damage in a mouse model of neuroendoscopy-induced brain injury. We investigated neurological function and inflammation following a 2-week treatment with osthole. The results suggest a new strategy for restoring neuronal function and improving long-term patient outcome after neuroendoscopy.

## Materials and methods

### Preparation of osthole

Osthole (catalog no. 110822-200305, purity >98 % (Fig. 1a) was purchased from the National Institute for the Control of Pharmaceutical and Biological Products (Beijing, China) and dissolved in dimethyl sulfoxide (DMSO; <0.1 %), and stored at 4 °C [20, 21].

### Animals, surgical procedures, and osthole administration

Adult C57BL/6J mice aged 3–4 months were housed under a 12:12-h light/dark cycle, with free access to food and water. Animal procedures conformed to guidelines set by Liaoning University of Traditional Chinese Medicine Institutional Animal Care and Use Committee, which are in accordance with those set by the National Institutes of Health (Bethesda, MD, USA). A mouse model of stab wound injury, as previously described in [29–31] with slight modifications, is created to mimic the neuroendoscopy procedure. Briefly, mice were anesthetized with ketamine/xylazine solution (50 mg/kg ketamine and 7.5 mg/kg xylazine in 0.9 % NaCl solution) and placed in a stereotaxic frame (ST-5ND-B, Chengdu, China). The head was shaved and the skin was disinfected with iodine/alcohol; body temperature was maintained at 37 °C throughout the surgical procedure using a heating pad [32]. A midline incision was made through the scalp and the skin was retracted. A hole was made over the left cerebral hemisphere using a dental drill until the dura was exposed. A 20-gauge, 1.1-mm-diameter needle with a rigid core (BD Nexiva Closed IV Catheter System; BD Biosciences, Franklin Lakes, NJ, USA) was inserted at 2.5 mm lateral to the midline, 2.5 mm posterior to the lambdoidal suture, and at a depth of 2.5 mm from the surface of the brain (Fig. 1b). A blunt catheter needle with a sharp core was inserted into the mouse brain. Upon reaching the target area, the needle was left in place for 20 min while the core was removed immediately. This procedure was designed to mimic neuroendoscopy. The injury site was then covered with sterile bone wax, the skin incision was closed with sutures, and the mouse was allowed to recover in its cage [33].

Mice were randomly divided into five groups: groups 1–3 (SWI+Ost) were administered osthole by i.p. injection 30 min after surgery at 10, 20, and 30 mg/kg, respectively, dissolved in 0.1 % DMSO and phosphate buffered saline (PBS) [11, 27] followed by once daily injections for the next 14 days (*n* = 30, 30, and 54 for groups 1, 2, and 3, respectively); mice in the SWI control group were given 0.1 % DMSO in PBS by i.p. injection (200 μl) each day for 14 days (*n* = 54), and naive C57BL/6J mice were used as controls (*n* = 54). Mice were sacrificed between 3 and 21 days post-injury (dpi) for analyses.

### Assessment of neurological function

Neurological function was assessed with a modified neurological severity score (NSS) at 12 h and 3, 7, 14, and 21 dpi, as previously described (*n* = 6 per group for each time point) [29, 34, 35]. The evaluation consisted of motor (muscle status and abnormal movement), sensory (visual, tactile, and proprioceptive), reflex, and balance tests, with results measured on a scale of 0–18 (0 = normal, 1–6 = mild injury, 7–12 = mean-moderate injury; 13–18 = severe injury, and 18 = maximal deficit) and the total score representing the sum of all test scores. One point was awarded for the inability to perform a test or lack of a tested reflex; therefore, a higher score indicated a greater degree of injury. The test was administered by blinded, trained investigators, and mice were familiarized with the testing environment before being subjected to brain injury.

### Measurement of brain water content

Brain water content was measured 72 h after SWI (*n* = 6 per group). Following anesthesia and decapitation, brains were dissected, separated along the midline, and the cerebellum was removed. Ipsilateral hemisphere wet weight was obtained on a pre-weighed piece of aluminum foil; after drying in an electric oven at 100 °C for 24 h, the percent water was calculated as (wet weight − dry weight)/(wet weight) [11, 36, 37].

### Hematoxylin and eosin staining and measurement of lesion size

At 3, 7, 14, and 21 dpi, mice were anesthetized and transcardially perfused with 4 % paraformaldehyde in cold phosphate buffer (*n* = 6 per group for each time point). Brains were immediately dissected and fixed in 10 % buffered formalin and embedded in paraffin. Serial coronal section 10 μm thick were cut in a direction parallel to the needle penetration line at 100-μm intervals so as to cover the entire lesion site. Sections were mounted on glass slides for hematoxylin and eosin (H & E) staining [37, 38] and visualized on a Nikon Eclipse E800 microscope (Tokyo, Japan) with a digital camera. Changes in cytoarchitecture were analyzed, and the size of the wound cavity was measured in each section by tracing a line across the top and along the edge of the tissue lining the lesion with ImageJ software (National Institutes of Health) [13, 18]. Measurements were taken from six sections per mouse from six mice at each time point [30, 39, 40].

### Immunocytochemistry

Brains were flash-frozen in cold isopentane on dry ice immediately following perfusion and stored at −80 °C. Serial frozen sections were cut at a thickness of 8 μm on a cryostat microtome (Leica, Nussloch, Germany) and fixed with 4 % paraformaldehyde in PBS for 30 min. After washing twice in PBS, endogenous peroxidase activity was quenched by incubation in 3 % hydrogen peroxide/0.1 % Triton X-100 for 15 min at room temperature, followed by two washes in PBS and blocking with 10 % goat serum for 30 min. Sections were incubated at 4 °C overnight with antibodies against the following proteins: neurofilament (NF)-M, neuronal nuclei (NeuN), glial fibrillary acidic protein (GFAP) (all at 1:150, from StemCell Technologies, Vancouver, Canada), ionized calcium-binding adaptor molecule (Iba)-1 (1:100), caspase-3 (1:150), and myeloperoxidase (MPO) (1:150) (all from Abcam, Cambridge, MA, USA). After three PBS washes, sections were incubated with appropriate fluorescein isothiocyanate- or Cy3-conjugated secondary antibodies (1:200; Jackson ImmunoResearch Lab, West Grove, PA, USA) for 60 min at room temperature and counterstained with 4′6-diamidino-2-phenylindole (DAPI), followed by three PBS washes. Sections were mounted with mounting medium (Vector Laboratories, Burlingame, CA, USA) and visualized using the Nikon Eclipse E800 microscope.

Cells expressing specific antigen were counted using ImageJ software [20, 31, 41–45] in six non-adjacent brain sections from six mice per group in five digital images of each section acquired with the same exposure parameters. The expression level was measured by pixel intensity as previously reported [44, 45]. Pixel intensity was measured from areas immediately surrounding the lesion (Fig. 1b). The total number of DAPI+ nuclei was similar between measured areas (*n* = 6 for each test).

### Terminal deoxynucleotidyl transferase dUTP nick end labeling (TUNEL)

Brain sections from mice at 7 dpi were analyzed for apoptotic cells using the Fluorescence In Situ Cell Death Detection kit (Roche, Chicago, IL, USA) according to the manufacturer’s instructions (*n* = 6 per group). The number of TUNEL-positive cells in each section in areas surrounding the lesion was counted in six sections per mouse and six mice per group using ImageJ software [9, 29, 39].

### Quantitative real-time (RT-) PCR

Brains were collected at 3 and 7 dpi (*n* = 6 per group for each time point), and punch biopsies (diameter: 5 mm) of the injured cortex were obtained. Tissue samples were rinsed with diethylpyrocarbonate-treated water, placed in 2-ml Eppendorf tubes, and stored at −80 °C for RT-PCR. Total RNA was extracted from the tissue with TRIzol reagent and reverse transcribed to cDNA using a RevertAid First Strand cDNA Synthesis kit (Thermo Scientific, Vilnius, Lithuania) [46]. The PCR reaction (35 cycles) was carried out using a DreamTaq Green PCR Master Mix Kit (Thermo Scientific). Quantitative RT-PCR was performed using the following forward and reverse primer sets designed using Premier Biosoft 5 (Palo Alto, CA, USA): Bax, 5′-CTG ACA TGT TTT CTG ACG GC-3′ and 5′-TCA GCC CAT CTT CTT CCA GA-3′; Bcl-2, 5′-CGC TGG GAG AAC AGG GTA-3′ and 5′-GGG CTG GGA GGA GAA GAT-3′; caspase-3, 5′-AGA TAC CGG TGG AGG CTG ACT-3′ and 5′-TCT TTC GTG AGC ATG GAC ACA-3′; IL-6, 5′-AGC CAG AGT CCT TCA GAG AG-3′ and 5′-TCC TTA GCC ACT CCT TCT GT-3′; and β-actin (control), 5′-GGG AAA TCG TGC GTG ACA T-3′ and 5′-TCA GGA GGA GCA ATG ATC TTG-3′. Products were resolved by 1.5 % agarose gel electrophoresis with ethidium bromide staining. The mRNA level of IL-6 was detected at 3 dpi, and those of Bax, Bcl-2, and caspase-3 were detected at 7 dpi. Quantitative analysis was performed using a Tanon 4100 Gel Imaging System (Tanon Science & Technology Co., Shanghai, China).

### Analysis of cytokine levels by enzyme-linked immunosorbent assay (ELISA)

Brains were collected at 3 dpi, and punch biopsies (diameter: 5 mm) of the injured cortex were obtained and stored at −80 °C until use (*n* = 6). Brain homogenates were centrifuged at 4 °C and 15,000 rpm for 20 min, and the supernatant was transferred to an Eppendorf tube. IL-6 and TNF-α levels in each sample were measured using ELISA kits (R&D Systems, Minneapolis, MN, USA) following the manufacturer’s instructions on a fluorescent plate reader at 450 nm [11].

### Statistical analysis

Data were analyzed using SPSS version 13.0 (SPSS, Chicago, IL, USA) and are presented as mean ± standard deviation. Differences between groups were assessed by one-way analysis of variance, and post-hoc multiple comparisons were carried out with the Student-Newman-Keuls test. *P* < 0.05 was considered statistically significant.

## Results

In this study, we used naive (uninjured) mice as controls rather than those subjected to craniotomy. It was previously shown that craniotomy is equivalent to a minor injury in terms of the acute inflammatory response that is induced [9, 47]; mice that underwent craniotomy had similar numbers of GFAP-positive astrocytes to those that experienced moderate cortical impact injury [48].

### Osthole treatment improves neurological function after SWI

**Fig. 2:**
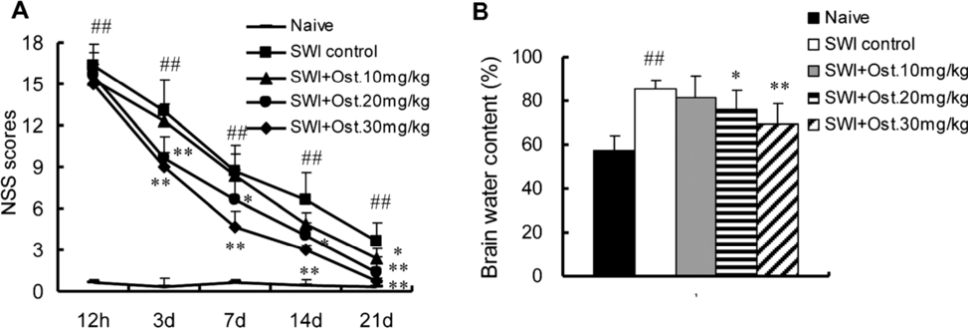
Effect of osthole on NSS and brain water content. **a** Neurological function was assigned an NSS at 12 h, 3, 7, 14, and 21 dpi. Treatment with osthole (20 or 30 mg/kg/day) lowered NSS from 3 to 21 dpi relative to untreated controls. There was no difference in the scores between the two groups at a concentration of 10 mg/kg/day. **b** Brain water content of injured hemispheres measured at 3 dpi. Water content was higher in the untreated SWI group than in naive controls. Osthole treatment reduced water content in a dose-dependent manner (*n* = 6 per group). Data represent mean ± SD. ^##^ *P* < 0.01 vs. naive control; *P < 0.05, **P < 0.01, vs. SWI control

Various concentrations of osthole (10, 20, or 30 mg/kg/day) were tested to determine whether it can reduce inflammation and promote neurological recovery after SWI. NSS was evaluated from 12 h to 21 dpi. SWI mice had impaired neurological function as compared to naïve control mice, as evidenced by the increased NSS. Osthole treatment lowered the NSS from 3 to 21 days in a dose-dependent manner; the most significant decrease was observed with 30 mg/kg osthole (0.77 ± 0.15 vs. 3.67 ± 1.28 in SWI controls at 21 dpi; *P* < 0.01) (Fig. 2a).

### Increase in brain water content caused by injury is reversed by osthole treatment

Cerebral edema is caused by damage to the blood-brain barrier (BBB) after traumatic injury, and the resultant increase in brain water content is a marker of brain damage [12]. Brain edema worsened over time, peaking at 72 h before declining thereafter [49]. At 3 dpi, brain water content was higher in the SWI than in the naïve control group (85.27 % ± 4.32 % vs. 57.56 % ± 6.45 %) (Fig. 2b); this was reduced in a dose-dependent manner by treatment with osthole (69.45 ± 9.26; *P* < 0.01 vs. SWI control).

### Osthole accelerates the closure of the brain cavity resulting from injury

**Fig. 3:**
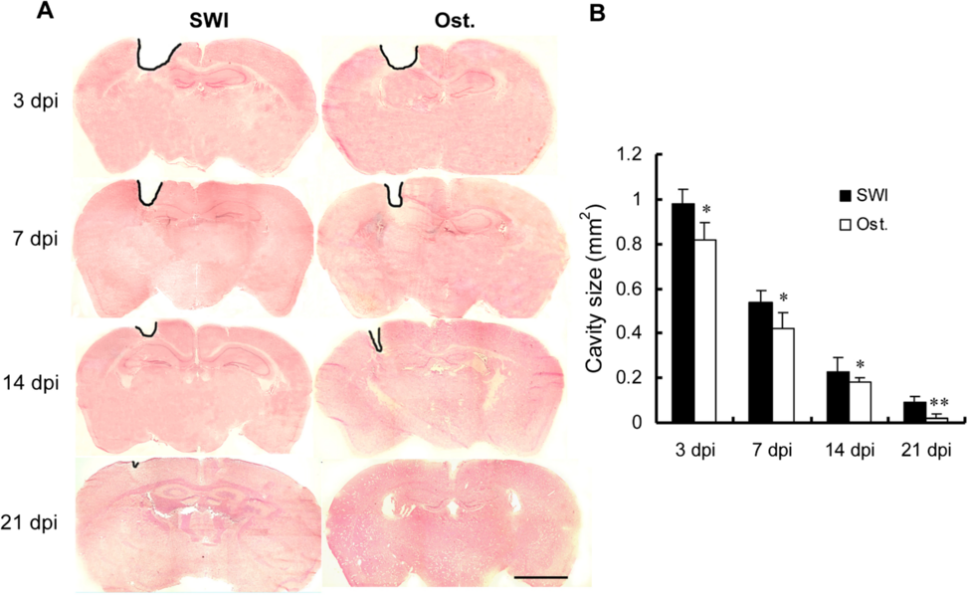
Osthole treatment reduces wound cavity size. **a** Representative images of hematoxylin and eosin-stained coronal sections at 3, 7, 14, and 21 dpi. The lesioned cortex is *outlined in black*. **b** Wound cavity area measurements. The area was smaller in osthole-treated mice than in untreated controls from 3 to 21 dpi. Measurements were obtained from six sections per mouse separated by 100 μm across the lesion, with six mice analyzed per time point. Data represent mean ± SD. *P < 0.05, **P < 0.01, vs. SWI control. *Scale bar*, 2.5 mm

H & E staining revealed damage to cortical tissue due to needle penetration. Figure 3a shows representative brain sections at different time points after injury. The cavity was largest at 3 dpi and shrank over time as the tissue healed: in osthole-treated and untreated mice, cavity size was smaller at 21 than at 3 dpi. In mice treated with osthole (30 mg/kg), the rate of cavity closure was accelerated, with a significant reduction in cavity size at each time point after injury (*P* < 0.01) (Fig. 3a, b). By 21 dpi, the lesion had closed in 4/6 osthole-treated mice vs. 2/6 untreated controls.

### Osthole attenuates the inflammatory response in the injured brain

**Fig. 4:**
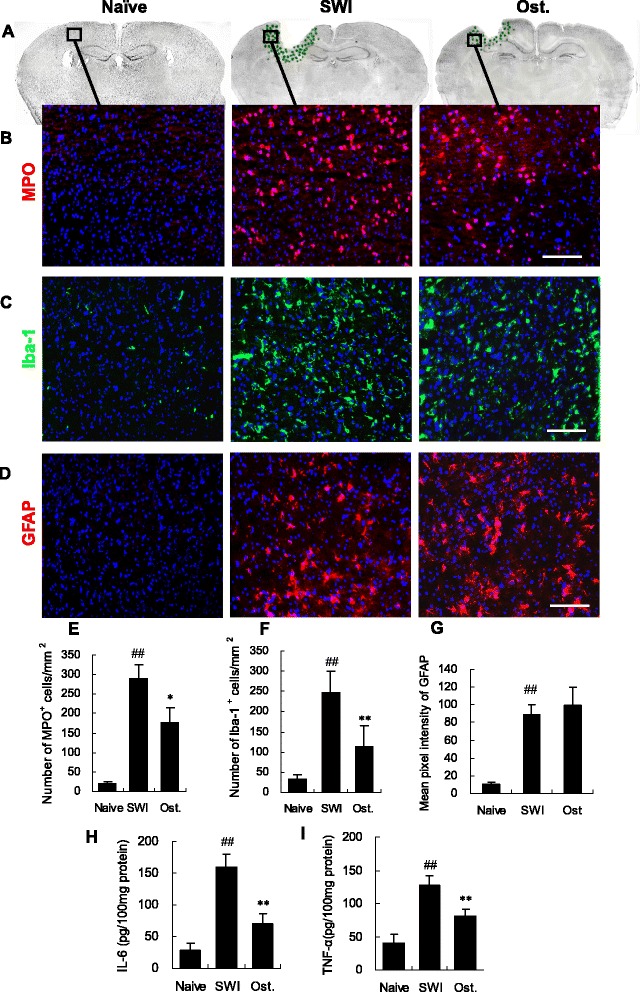
Osthole treatment reduces inflammatory cell infiltration and proinflammtory cytokines levels. **a** Spatial distribution of inflammatory cells around the lesion at 3 dpi. **b**–**d** MPO+, Iba-1+, and GFAP+ cells, as detected by immunohistochemistry within a single field (*box*) in the lesioned cortex. Macrophages/microglia (Iba-1+, *green*), neutrophils (MPO+, *red*), and astrocytes (GFAP+, *red*) are shown along with nuclear DAPI staining (*blue*). **e**, **f** Quantitative analysis of inflammatory cells as a function of total cell number (DAPI+). **g** Quantitative analysis of GFAP expression, as determined by measuring GFAP immunoreactivity pixel intensity. Regions immediately surrounding the lesion (as shown in panel **a**) were examined using ImageJ software. *Scale bar*, 50 μm in B–D. **h**, **i** IL-6 and TNF-α levels, as assessed by ELISA. Data represent mean ± SD (*n* = 6 per group). ^##^ *P* < 0.01 vs. naive control; *P < 0.05, **P < 0.01, vs. SWI control

Neuroinflammation following brain injury is characterized by macrophage and neutrophil infiltration and microglia activation [30, 50]. Proinflammatory cytokines such as TNF-α and IL-6 are mainly produced by microglia, which in turn activate glia, further stimulating cytokine production and astrogliosis [11, 14, 51]. Suppressing microglia activation can therefore reduce inflammation and improve recovery from injury [17]. To assess the role of osthole in the inflammatory response, we quantified the numbers of Iba-1+ macrophages/microglia, MPO+ neutrophils, and the fluorescence intensity of GFAP+ astrocytes in the lesioned cortex and measured the levels of inflammation-associated cytokines in cortical tissue homogenates by ELISA at 3 dpi. Inflammation-associated cells were predominantly localized around the lesion following injury (Fig. 4a); however, there were fewer neutrophils and macrophages/microglia in osthole-treated as compared to SWI control mice (Iba-1+: 115.33 ± 47.96 vs. 247.83 ± 52.29 cells/mm^2^; MPO+: 176.00 ± 45. 4 vs. 290.67 ± 34.89 cells/mm^2^, *P* < 0.01) (Fig. 4b, c, e, f). In contrast, GFAP immunoreactivity in astrocytes was unaltered by osthole treatment (Fig. 4d, g). Additionally, levels of the proinflammatory cytokines IL-6 and TNF-α were reduced in osthole-treated relative to untreated control animals (IL-6: 70.73 ± 15.59 vs. 160.09 ± 19.59 pg/100 mg; TNF-α: 80.93 ± 9.90 vs. 127.08 ± 15.36 pg/100 mg; *P* < 0.01) (Fig. 4h, i). These results suggest that osthole suppresses trauma-induced inflammation by inhibiting microglia activation and neutrophil infiltration as well as the release of proinflammatory cytokines that can cause secondary damage to the brain after injury.

### Osthole promotes neuronal survival and reduces apoptosis in the injured brain

**Fig. 5:**
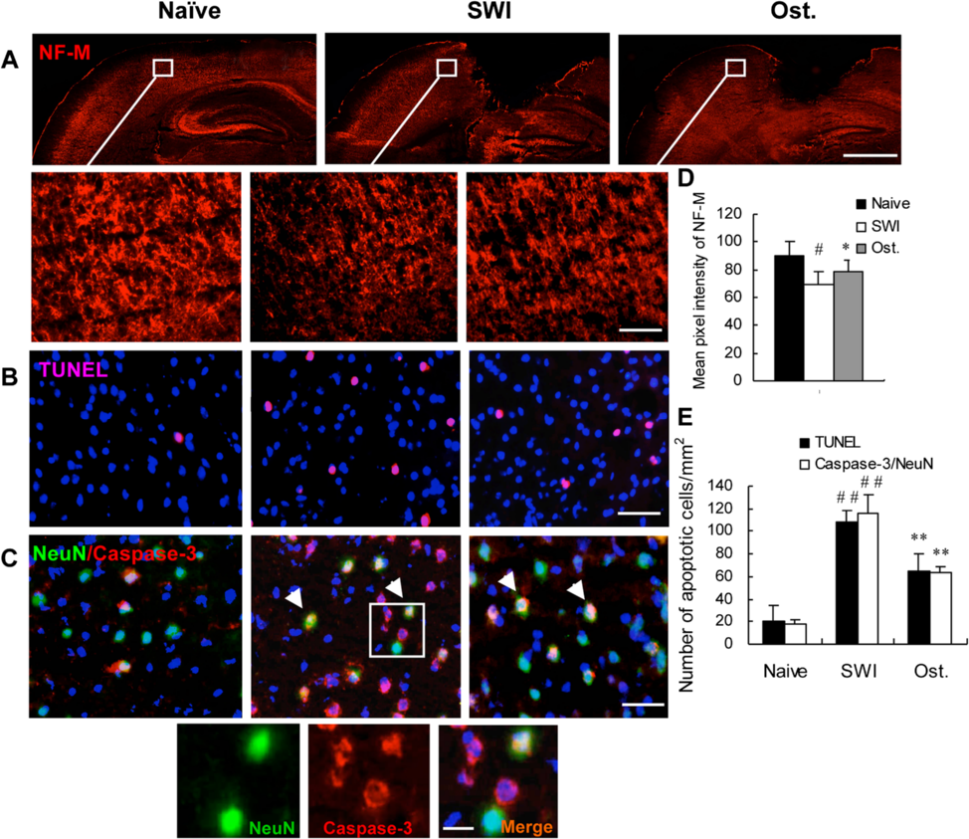
Osthole treatment promotes neuronal survival and inhibits apoptosis in the lesioned brain. **a**, **d** Neuronal survival in the lesioned cortex of mice was detected at 7 dpi by immunocytochemistry with antibodies against NF-M and NeuN. NF-M expression around the lesion was quantified by measuring pixel intensity of NF-M immunoreactivity using ImageJ software. *Areas in boxes* are shown at higher magnification in the insets. *Scale bar*, 250 μm in **a**, 25 μm in the insets. **b** Neuronal apoptosis as determined by the TUNEL assay. **c** Apoptotic neurons were labeled with anti-NeuN (*green*) and anti-caspase-3 (*red*) antibodies at 7 dpi; double positive cells indicated by arrows are shown at higher magnification in the insets. *Scale bar*, 25 μm in **b** and **c**, 10 μm in the insets. **e** Quantitative analysis of TUNEL-positive and NeuN/caspase-3 double positive cells. Nuclei were stained with DAPI (*blue*). Areas surrounding the lesion (Fig. 1b) were examined. Data represent mean ± SD (*n* = 6 per group). ^##^ *P* < 0.01 vs. naive control; *P < 0.05, **P < 0.01, vs. SWI control

To evaluate the effects of osthole treatment at a cellular level, brain sections at 7 dpi were examined for NF-M expression (Fig. 5a, d) and apoptotic neurons were detected by the TUNEL assay (Fig. 5b, e) and by double labeling with antibodies against caspase-3 and NeuN (Fig. 5c, e). SWI resulted in decreased expression of NF-M, representing a loss of axons. On the other hand, osthole treatment increased NF-M immunoreactivity in the lesion area as compared to untreated controls (fluorescence intensity: 78.5 ± 8.36 vs. 69.45 ± 7.93; *P* < 0.05) (Fig. 5a, d) and decreased the number of TUNEL+ and NeuN+/caspase-3+ apoptotic neurons (TUNEL+: 65.00 ± 15.56 vs. 108.00 ± 9.90 cells/mm^2^; caspase-3+/NeuN+: 64 ± 4.58 vs. 115.33 ± 17.63 cells/mm^2^; *P* < 0.05) (Fig. 5b, c, e). Thus, osthole promotes neuronal recovery by inhibiting apoptosis after SWI.

**Fig. 6:**
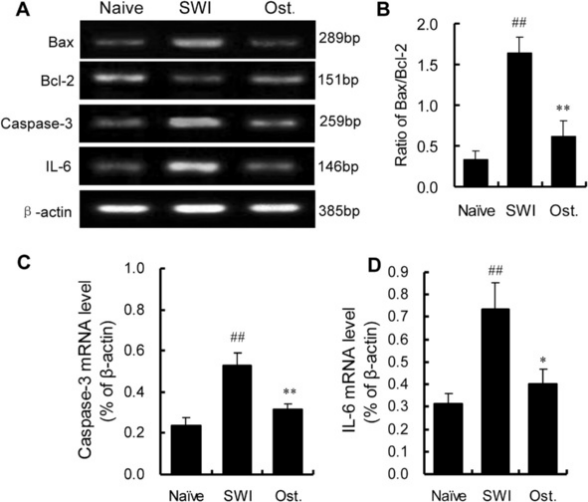
Effect of osthole on gene expression in the injured brain. IL-6 mRNA expression was examined at 3 dpi, and Bax, Bcl-2, and caspase-3 levels were examined at 7 dpi. **a** mRNA expression as detected by RT-PCR. **b** Quantitative analysis of Bax/Bcl-2 ratio. **c**, **d** Quantitative analysis of caspase-3 and IL-6 mRNA levels. Osthole treatment reduced IL-6 and caspase-3 levels as well as Bax/Bcl-2 ratio relative to untreated controls. Data represent mean ± SD (*n* = 6 per group). ^##^ *P* < 0.01 vs. naive control; *P < 0.05, **P < 0.01, vs. SWI control

To clarify the mechanisms underlying the anti-inflammatory and -apoptotic functions of osthole, we examined the levels of mRNA expression of the proinflammtory cytokine IL-6 and apoptotic factors Bax, Bcl-2 and caspase-3 by RT-PCR. SWI induced an increase in IL-6, Bax, and caspase-3 and a decrease in Bcl-2 transcript. Osthole treatment resulted in the downregulation of IL-6 and caspase-3 and reduced the ratio of Bax to Bcl-2 as compared to the untreated SWI group (IL-6: 0.40 ± 0.07 vs. 0.73 ± 0.12; caspase-3:0.34 ± 0.03 vs. 0.51 ± 0.06; Bax to Bcl-2 ratio: 0.62 ± 0.18 vs. 1.64 ± 0.20; *P* < 0.01) (Fig. 6a–d). These results suggest that osthole exerts anti-inflammatory effects by inhibiting IL-6 expression and suppresses apoptosis by reducing the Bax to Bcl-2 ratio and downregulating caspase-3 expression.

## Discussion

In this study, we mimicked the procedure of neuroendoscopy by using a needle to create a stab wound in the mouse brain and investigated whether osthole treatment can protect against secondary brain damage. Osthole treatment (30 mg/kg i.p. once daily for 14 days) reduced the number of microglia/macrophages in the brain parenchyma, decreased the number of peripheral infiltrating leukocytes at the lesion, reduced proinflammatory cytokine level, and inhibited apoptosis. These effects were exerted via downregulation of IL-6, TNF-α, and caspase-3 expression and a reduction in the ratio of Bax to Bcl-2. Thus, osthole can prevent secondary brain damage through anti-inflammatory as well as anti-apoptotic mechanisms.

Traumatic injury to the brain triggers an inflammatory response characterized by activation of microglia and invasion of peripheral macrophages, resulting in secondary tissue damage at the injury site [19, 52]. The lack of spontaneous tissue repair is due not only to the inability of neurons to proliferate but also to the lesion microenvironment, which contains many toxic factors [53, 54]. Accordingly, the goal of treatment is to suppress inflammation during the acute phase so as to improve the environment for and prevent secondary damage to surviving neurons.

Osthole has anti-inflammatory, anti-immunomodulatory, anti-apoptotic, and anti-oxidative stress as well as neurotrophic effects [20–24]. It also protects against cerebral ischemic injury by reducing oxidative stress injury and preserving the integrity of the BBB [55]. Our previous study showed that osthole treatment reduced clinical severity in EAE mice by suppressing the autoimmune response, decreasing IL-17 and IFN-γ production, and increasing neurotrophic support, thereby creating an environment favorable to neuronal survival, protecting the myelin sheath from demyelination, and promoting the repair of myelin/axons [20], as reported by others [24]. In the present study, mice treated with osthole exhibited a low NSS, which was accompanied by a significant reduction in brain water content and accelerated closure of the wound cavity as compared to untreated control animals. Moreover, osthole treatment following injury was also associated with a lower number of microglia/macrophages and neutrophils at the injury site and reduced levels of the proinflammatory cytokines IL-6 and TNF-α. The effects of osthole also included inhibition of apoptosis, which is mediated in part by caspase-3 activation [56–58]. Thus, our results suggest that osthole can prevent post-injury inflammation and apoptosis to reduce secondary injury to brain tissue, creating an environment conducive to functional recovery.

A previous study has shown that initial needle penetration results in an immediate loss of tissue and the formation of a cavity that gradually increases in size, reaching a maximum size 3 days after injury, at which time peak rates of apoptosis around the lesioned area and robust astroglial reactivity are also observed [10]. The extent of brain tissue damage was dependent on the mechanics of needle insertion; for example, high insertion force and speed produced greater tissue damage [32]. As expected, the cavity volume was larger than the diameter of needle that was used. The cavity shrank over time as the tissue healed, which was consistent with previous reports [59, 60].

NFs are the most abundant cytoskeletal protein in large myelinated axons [40, 61]; specifically, NF-M is important for the stabilization of mature axons [62]. We observed a decrease in NF-M expression in injured animals, indicating axonal loss. In contrast, mice treated with osthole showed increased NF-M expression, providing structural evidence for the effects of osthole in promoting neuronal restoration in the injured brain.

IL-6 is a major inducer of immune and inflammatory responses under conditions of injury [63] and enhances glutamate-mediated excitotoxicity in cerebellar granule cells in vitro and causes damage to the BBB in vivo [64]. Transgenic mice overexpressing IL-6 display gliosis, neuronal loss, and learning disabilities with prominent neurodegeneration [65]. Hence, excessive IL-6-mediated inflammation is likely involved in the unfavorable outcomes associated with SWI. Osthole treatment partly blocked the increase in *IL-6* gene expression resulting from injury, providing additional evidence that osthole suppresses neuroinflammation via downregulation of IL-6.

The *Bcl-2* family includes genes encoding the pro-apoptotic protein Bax and the anti-apoptotic protein Bcl-2 [66, 67]. Bcl-2 overexpression inhibits neuronal apoptosis and stimulates the recovery of neurological function [68, 69], while overexpressing Bax induces apoptosis [70]. Bax upregulation and Bcl-2 downregulation increases the Bax to Bcl-2 ratio; this may be directly associated with cytochrome c release [71] and increased expression of caspase-3, which induces apoptosis [72]. In the present study, osthole treatment reduced the Bax to Bcl-2 ratio and caspase-3 level that were elevated by SWI, providing insight into the mechanism underlying the anti-apoptotic effects of osthole.

## Conclusions

Osthole treatment conferred neuroprotection and reduced tissue damage in an experimental cortical SWI model, reducing secondary brain damage via anti-inflammatory and -apoptotic mechanisms. These findings demonstrate that osthole has therapeutic potential for reducing injury-induced neuroinflammation to improve long-term patient outcome after neuroendoscopic surgery.

## References

[1] Kelly PJ, Goerss SJ, Kall BA (1988). The stereotaxic retractor in computer-assisted stereotaxic microsurgery. Technical note. J Neurosurg.

[2] Nishihara T, Teraoka A, Morita A, Ueki K, Takai K, Kirino T (2000). A transparent sheath for endoscopic surgery and its application in surgical evacuation of spontaneous intracerebral hematomas. Technical note. J Neurosurg.

[3] Kassam AB, Engh JA, Mintz AH, Prevedello DM (2009). Completely endoscopic resection of intraparenchymal brain tumors. J Neurosurg.

[4] Waran V, Vairavan N, Sia SF, Abdullah B (2009). A new expandable cannula system for endoscopic evacuation of intraparenchymal hemorrhages. J Neurosurg.

[5] Ding D, Starke RM, Webster Crowley R, Liu KC. Endoport-assisted microsurgical resection of cerebral cavernous malformations. J Clin Neurosci. 2015; S0967-5868(15)00018-1. doi: 10.1016/j.jocn.2015.01.004. [Epub ahead of print]. 10.1016/j.jocn.2015.01.004 25769248

[6] Polikov VS, Tresco PA, Reichert WM (2005). Response of brain tissue to chronically implanted neural electrodes. J Neurosci Methods.

[7] Wagshul ME, Eide PK, Madsen JR (2011). The pulsating brain: a review of experimental and clinical studies of intracranial pulsatility. Fluids Barriers CNS.

[8] Wallenquist U, Holmqvist K, Hånell A, Marklund N, Hillered L, Forsberg-Nilsson K (2012). Ibuprofen attenuates the inflammatory response and allows formation of migratory neuroblasts from grafted stem cells after traumatic brain injury. Restor Neurol Neurosci.

[9] Bayir H, Kochanek PM, Clark RS (2003). Traumatic brain injury in infants and children: mechanisms of secondary damage and treatment in the intensive care unit. Crit Care Clin.

[10] Villapol S, Byrnes KR, Symes AJ (2014). Temporal dynamics of cerebral blood flow, cortical damage, apoptosis, astrocyte–vasculature interaction and astrogliosis in the pericontusional region after traumatic brain injury. Front Neurol.

[11] Xia L, Jiang ZL, Wang GH, Hu BY, Ke KF (2012). Treatment with ginseng total saponins reduces the secondary brain injury in rat after cortical impact. J Neurosci Res.

[12] Zhang R, Liu Y, Yan K, Chen L, Chen XR, Li P (2013). Anti-inflammatory and immunomodulatory mechanisms of mesenchymal stem cell transplantation in experimental traumatic brain injury. J Neuroinflammation.

[13] Helmy A, Carpenter KL, Menon DK, Pickard JD, Hutchinson PJ (2011). The cytokine response to human traumatic brain injury: temporal profiles and evidence for cerebral parenchymal production. J Cereb Blood Flow Metab.

[14] Ziebell JM, Morganti-Kossmann MC (2010). Involvement of pro- and anti-inflammatory cytokines and chemokines in the pathophysiology of traumatic brain injury. Neurotherapeutics.

[15] Rhodes J (2011). Peripheral immune cells in the pathology of traumatic brain injury?. Curr Opin Crit Care.

[16] Lucas SM, Rothwell NJ, Gibson RM (2006). The role of inflammation in CNS injury and disease. Br J Pharmacol.

[17] Helmy A, De Simoni MG, Guilfoyle MR, Carpenter KL, Hutchinson PJ (2011). Cytokines and innate inflammation in the pathogenesis of human traumatic brain injury. Prog Neurobiol.

[18] D’Avila JC, Lam TI, Bingham D, Shi J, Won SJ, Kauppinen TM (2012). Microglial activation induced by brain trauma is suppressed by post-injury treatment with a PARP inhibitor. J Neuroinflammation.

[19] Su Z, Yuan Y, Cao L, Zhu Y, Gao L, Qiu Y (2010). Triptolide promotes spinal cord repair by inhibiting astrogliosis and inflammation. Glia.

[20] Gao Z, Wen Q, Xia Y, Yang J, Gao P, Zhang N (2014). Osthole augments therapeutic efficiency of neural stem cells-based therapy in experimental autoimmune encephalomyelitis. J Pharmacol Sci.

[21] Hu Y, Wen Q, Liang W, Kang T, Ren L, Zhang N (2013). Osthole reverses beta-amyloid peptide cytotoxicity on neural cells by enhancing cyclic AMP response element-binding protein phosphorylation. Biol Pharm Bull.

[22] Chen T, Liu W, Chao X, Qu Y, Zhang L, Luo P (2011). Neuroprotective effect of osthole against oxygen and glucose deprivation in rat cortical neurons: involvement ofmitogen-activated protein kinase pathway. Neuroscience.

[23] Ji HJ, Hu JF, Wang YH, Chen XY, Zhou R, Chen NH (2010). Osthole improves chronic cerebral hypoperfusion induced cognitive deficits and neuronal damage in hippocampus. Eur J Pharmacol.

[24] Chao X, Zhou J, Chen T, Liu W, Dong W, Qu Y (2010). Neuroprotective effect of osthole against acute ischemic stroke on middle cerebral ischemia occlusion in rats. Brain Res.

[25] Okamoto T, Yoshida S, Kobayashi T, Okabe S (2001). Inhibition of concanavalin A-induced mice hepatitis by coumarin derivatives. Jpn J Pharmacol.

[26] Yang LL, Wang MC, Chen LG, Wang CC (2003). Cytotoxic activity of coumarins from the fruits of Cnidium monnieri on leukemia cell lines. Planta Med.

[27] You L, Feng S, An R, Wang X (2009). Osthole: a promising lead compound for drug discovery from a traditional Chinese medicine (TCM). Nat Prod Commun.

[28] Chen X, Pi R, Zou Y, Liu M, Ma X, Jiang Y (2010). Attenuation of experimental autoimmune encephalomyelitis in C57 BL/6 mice by osthole, a natural coumarin. Eur J Pharmacol.

[29] Liu SJ, Zou Y, Belegu V, Lv LY, Lin N, Wang TY (2014). Co-grafting of neural stem cells with olfactory en sheathing cells promotes neuronal restoration in traumatic brain injury with an anti-inflammatory mechanism. J Neuroinflammation.

[30] Wang Y, Moges H, Bharucha Y, Symes A (2007). Smad3 null mice display more rapid woun closure and reduced scar formation after a stab wound to the cerebralcortex. Exp Neurol.

[31] Takarada-Iemata M, Kezuka D, Takeichi T, Ikawa M, Hattori T, Kitao Y (2014). Deletion of N-myc downstream-regulated gene 2 attenuates reactive astrogliosis and inflammatory response in a mouse model of cortical stab injury. J Neurochem.

[32] Casanova F, Carney PR, Sarntinoranont M (2014). In vivo evaluation of needle force and friction stress during insertion at varying insertion speed into the brain. J Neurosci Methods.

[33] Villapol S, Wang Y, Adams M, Symes AJ (2013). Smad3 deficiency increases cortical and hippocampal neuronal loss following traumatic brain injury. Exp Neurol.

[34] Hirjak D, Wolf RC, Stieltjes B, Hauser T, Seidl U, Thiemann U (2013). Neurological soft signs and brainstem morphology in first-episode schizophrenia. Neuropsychobiology.

[35] Lu M, Chen J, Lu D, Yi L, Mahmood A, Chopp M (2003). Global test statistics for treatment effect of stroke and traumatic brain injury in rats with administration of bone marrow stromal cells. J Neurosci Methods.

[36] Lee ST, Chu K, Jung KH, Kim SJ, Kim DH, Kang KM (2008). Anti-inflammatory mechanism of intravascular neural stem cell transplantation in haemorrhagic stroke. Brain.

[37] Taya K, Marmarou CR, Okuno K, Prieto R, Marmarou A (2010). Effect of secondary insults upon aquaporin-4 water channels following experimental cortical contusion in rats. J Neurotrauma.

[38] Bitto A, Polito F, Irrera N, Calò M, Spaccapelo L, Marini HR (2012). Protective effects of melanocortins on short-term changes in a rat model of traumatic brain injury. Crit Care Med.

[39] Yin F, Guo L, Meng CY, Liu YJ, Lu RF, Li P (2014). Transplantation of mesenchymal stem cells exerts anti-apoptotic effects in adult rats after spinal cord ischemia-reperfusion injury. Brain Res.

[40] Hozumi I, Chiu FC, Norton WT (1990). Biochemical and immunocytochemical changes in glial fibrillary acidic protein after stab wounds. Brain Res.

[41] Yang J, Yan Y, Xia Y, Kang T, Li X, Ciric B (2014). Neurotrophin 3 transduction augments remyelinating and immunomodulatory capacity of neural stem cells. Mol Ther.

[42] Zhang N, Wen Q, Ren L, Liang W, Xia Y, Zhang X (2013). Neuroprotective effect of arctigenin via upregulation of P-CREB in mouse primary neurons and human SH-SY5Y neuroblastoma cells. Int J Mol Sci.

[43] Zhang N, Kang T, Xia Y, Wen Q, Zhang X, Li H (2012). Effects of salvianolic acid B on survival, self-renewal and neuronal differentiation of bone marrow derived neural stem cells. Eur J Pharmacol.

[44] Yang J, Jiang Z, Fitzgerald DC, Ma C, Yu S, Li H (2009). Adult neural stem cells expressing IL-10 confer potent immunomodulation and remyelination in experimental autoimmune encephalitis. J Clin Invest.

[45] Yang J, Yan Y, Ciric B, Yu S, Guan Y, Xu H (2010). Evaluation of bone marrow- and brain-derived neural stem cells in therapy of central nervous system autoimmunity. Am J Pathol.

[46] Yang J, Bridges K, Chen KY, Liu AY (2008). Riluzole increases the amount of latent HSF1 for an amplified heat shock response and cytoprotection. PLoS One.

[47] Cole JT, Yarnel A, Kean WS, Gold E, Lewis B, Ren M (2011). Craniotomy: true sham for traumatic brain injury, or a sham of a sham?. J Neurotrauma.

[48] Susarla BT, Villapol S, Yi JH, Geller HM, Symes AJ (2014). Temporal patterns of cortical proliferation of glial cell populations after traumatic brain injury in mice. ASN Neuro.

[49] Baskaya MK, Rao AM, Dogan A, Donaldson D, Dempsey RJ (1997). The biphasic opening of the blood-brain barrier in the cortex and hippocampus after traumatic brain injury in rats. Neurosci Lett.

[50] Clausen F, Hånell A, Björk M, Hillered L, Mir AK, Gram H (2009). Neutralization of interleukin-1beta modifies the inflammatory response and improves histological and cognitive outcome following traumatic brain injury in mice. Eur J Neurosci.

[51] Lau LT, Yu AC (2001). Astrocytes produce and release interleukin-1, interleukin-6, tumor necrosis factor alpha and interferon-gamma following traumatic and metabolic injury. J Neurotrauma.

[52] Kawano H, Kimura-Kuroda J, Komuta Y, Yoshioka N, Li HP, Kawamura K (2012). Role of the lesion scar in the response to damage and repair of the central nervous system. Cell Tissue Res.

[53] Fleming JC, Norenberg MD, Ramsay DA, Dekaban GA, Marcillo AE, Saenz AD (2006). The cellular inflammatory response in human spinal cords after injury. Brain.

[54] Yiu G, He Z (2006). Glial inhibition of CNS axon regeneration. Nat Rev Neurosci.

[55] Chen Z, Mao X, Liu A, Gao X, Chen X, Ye M (2015). Osthole, a natural coumarin improves cognitive impairments and BBB dysfunction after transient global brain ischemia in C57 BL/6J mice: involvement of Nrf2 pathway. Neurochem Res.

[56] Schaible EV, Steinsträßer A, Jahn-Eimermacher A, Luh C, Sebastiani A, Kornes F (2013). Single administration of tripeptide α-MSH(11–13) attenuates brain damage by reduced inflammation and apoptosis after experimental traumatic brain injury in mice. PLoS One.

[57] Springer JE (2002). Apoptotic cell death following traumatic injury to the central nervous system. J Biochem Mol Biol.

[58] Stoica BA, Faden AI (2010). Cell death mechanisms and modulation in traumatic brain injury. Neurotherapeutics.

[59] Casanova F, Carney PR, Sarntinoranont M (2014). Effect of needle insertion speed on tissue injury, stress, and backflow distribution for convection-enhanced delivery in the rat brain. PLoS One.

[60] Bjornsson CS, Oh SJ, Al-Loafahi YA, Lim YJ, Smith KL (2006). Effects of insertion conditions on tissue strain and vascular damage during neuroprosthetic device insertion. J Neural Eng.

[61] Perrot R, Berges R, Bocquet A, Eyer J (2008). Review of the multiple aspects of neurofilament functions, and their possible contribution to neurodegeneration. Mol Neurobiol.

[62] Yabe JT, Wang FS, Chylinski T, Katchmar T, Shea TB (2001). Selective accumulation of the high molecular weight neurofilament subunit within the distal region of growing axonal neurites. Cell Motil Cytoskeleton.

[63] Wang H, Wang K, Zhong X, Dai Y, Qiu W, Wu A (2012). Notable increased cerebrospinal fluid levels of soluble interleukin-6 receptors in neuromyelitis optica. Neuroimmunomodulation.

[64] Farkas G, Márton J, Nagy Z, Mándi Y, Takács T, Deli MA (1998). Experimental acute pancreatitis results in increased blood-brain barrier permeability in the rat: a potential role for tumor necrosis factor and interleukin 6. Neurosci Lett.

[65] Xie F, Fang C, Schnittke N, Schwob JE, Ding X (2013). Mechanisms of permanent loss of olfactory receptor neurons induced by the herbicide 2,6-dichlorobenzonitrile: effects on stem cells and noninvolvement of acute induction of the inflammatory cytokine IL-6. Toxicol Appl Pharmacol.

[66] Kotipatruni RR, Dasari VR, Veeravalli KK, Dinh DH, Fassett D, Rao JS (2011). p53- and Bax-mediated apoptosis in injured rat spinal cord. Neurochem Res.

[67] Youle RJ, Strasser A (2008). The BCL-2 protein family: opposing activities that mediate cell death. Nat Rev Mol Cell Biol.

[68] Fan J, Xu G, Nagel DJ, Hua Z, Zhang N, Yin G (2010). A model of ischemia and reperfusion increases JNK activity, inhibits the association of BAD and 14-3-3, and induces apoptosis of rabbit spinal neurocytes. Neurosci Lett.

[69] Allsopp TE, Wyatt S, Paterson HF, Davies AM (1993). The proto-oncogene bcl-2 can selectively rescue neurotrophic factor-dependent neurons from apoptosis. Cell.

[70] Gross A, Jockel J, Wei MC, Korsmeyer SJ (1998). Enforced dimerization of BAX results in its translocation, mitochondrial dysfunction and apoptosis. EMBO J.

[71] Adams JM, Cory S (1998). The Bcl-2 protein family: arbiters of cell survival. Science.

[72] Li P, Nijhawan D, Budihardjo I, Srinivasula SM, Ahmad M, Alnemri ES, et al. Cytochrome c and dATP-dependent formation of Apaf-1/caspase-9 complex initiates an apoptotic proteasecascade. Cell. 1997;91(4):479–89. 10.1016/s0092-8674(00)80434-1 9390557

